# Outcomes of revision surgery for idiopathic macular hole after failed primary vitrectomy

**DOI:** 10.3389/fmed.2023.1169776

**Published:** 2023-07-27

**Authors:** Yunhong Shi, Lujia Feng, Yangyang Li, Zhihao Jiang, Dong Fang, Xiaotong Han, Lanhua Wang, Yantao Wei, Ting Zhang, Shaochong Zhang

**Affiliations:** ^1^State Key Laboratory of Ophthalmology, Zhongshan Ophthalmic Center, Sun Yat-sen University, Guangdong Provincial Key Laboratory of Ophthalmology and Visual Science, Guangdong Provincial Clinical Research Center for Ocular Diseases, Guangzhou, China; ^2^Shenzhen Eye Hospital, Jinan University, Shenzhen Eye Institute, Shenzhen, China

**Keywords:** persistent idiopathic macular hole, revision procedure, optical coherence tomography, visual acuity, pars plana vitrectomy

## Abstract

Persistent idiopathic macular hole (PIMH), the occurrence of idiopathic macular holes that have failed to close after standard pars plana vitrectomy (PPV) with internal limiting membrane (ILM) peeling, has become a global health threat to the aging population. Because postoperative anatomic closure or restoration of visual acuity is more difficult to achieve in PIMH, surgical approaches that would yield the best outcomes remain to be elucidated. On paper, extended ILM peeling combined with silicone oil (SiO) tamponade is believed to be a feasible option for excellent macular hole closure. However, no studies on this combined treatment for PIMH is compared with simple air tamponade have been conducted. Thus, in this retrospective case series, we used spectral-domain optical coherence tomography (SD-OCT) and other technologies to investigate real-world evidence for the anatomical and functional outcomes of revisional PPV with either SiO or air tamponade for failed primary idiopathic macular hole surgery. We included the records of 76 patients with PIMH who had SD-OCT examinations and best-corrected visual acuity (BCVA). Regression analysis was performed to find factors affecting PIMH fracture closure. Seventy-six participants were allocated to a SiO group (n = 21, with an extended ILM peeling and SiO tamponade) or an air group (n = 55, with extended ILM peeling and air tamponade). Anatomical success was achieved in 18 (85.7%) and 40 (72.7%) eyes in the SiO and air groups, respectively (*p* = 0.37). BCVA was significantly improved in both subgroups of closed PIMH (SiO group: *p* = 0.041; air group: *p* < 0.001). Minimum linear diameter (MLD) was closely related to the closure rate (OR, 1.0; 95% CI (0.985–0.999); *p* = 0.03). MLD = 650 μm seemed like a cut-off point for closure rate (MLD ≤ 650 μm vs. MLD > 650 μm; 88.4% vs. 52%, *p* = 0.002). In conclusion, we demonstrated that extended ILM peeling combined with SiO or air tamponade is effective in PIMH treatment. Moreover, though not statistically significant herein, the anatomic closure rate was better for silicone-operated eyes than for air-operated eyes. MLD is the best predictor of PIMH closure; MLD ≤ 650 μm could achieve a significantly higher closure rate.

## 1. Introduction

Since the initial publication by Kelly and Wendel describing vitrectomy surgery for idiopathic macular hole (iMH), the rate of successful macular hole closure has increased to over 90% (1, 2). However, the most common postoperative complication of iMH surgery is persistent iMH (PIMH). Because postoperative anatomic closure (52–80%) or restoration of visual acuity is more difficult to achieve in PIMH, it remains debatable which surgical approach is the best (3).

To increase the closure rate of PIMH, clinical researchers have focused more on completely relieving residual traction and prolonging effective tamponade to stimulate glial cell proliferation (4). Therefore, studies have described improvements in the standard surgical strategies, such as superior wide-base internal limiting membrane flap transposition (5), extended internal limiting membrane (ILM) peeling (6), use of an inverted ILM flap (7), transplantation of a neurosensory retina (8), and use of vitreous substitutes (9), as well as shown innovations in affiliation procedures to improve macular hole bridging, such as the use of whole blood or blood components (10). Air and silicone oil (SiO) are common tamponades. A series of reports have suggested the pleasantness of air tamponade effects in PIMH (11, 12). SiO, a long-acting vitreous substitute, is applied long-term in complicated vitreoretinal surgeries, and a recent meta-analysis showed its benefits in increasing the closure rate of large macular hole with or without retinal detachment (13). In addition, in recent years, enlarged ILM peeling has been widely used to treat various kinds of refractory macular holes because of its ability to relieve residual macular traction. Theoretically, the combined procedure of extended ILM peeling and SiO tamponade will achieve excellent iMH closure, but there are no reports on this kind of treatment for PIMH comparing it with simple air tamponade. Therefore, we conducted a retrospective case series to evaluate the effects of the two procedures, explore their indications, and determine the optimal intervention for relatively large PIMHs in a cohort of 76 patients.

## 2. Materials and methods

PIMH refers to the occurrence of iMH that have failed to close after standard pars plana vitrectomy (PPV) with ILM peeling. All patients underwent small-gauge (25–27 gauge) PPV with retrobulbar anesthesia, which was performed by experienced ophthalmologist, under monitored anesthetic care. Patients were allocated to either the SiO or air group at the discretion of the treating surgeon. Normally, by clinical experience, patients with a poor degree of adaptability, monophthalmia, or a planned flight were advised to undergo SiO tamponade.

### 2.1. Ethics statement

This study was approved by the Institutional Review Board of Zhongshan Ophthalmic Center, which is affiliated with Sun Yat-sen University (Guangzhou, China), and was performed in accordance with the World Medical Association’s Declaration of Helsinki. All extracted patient data were anonymized for analysis.

### 2.2. Inclusion and exclusion criteria

The inclusion criteria were the availability of high-quality spectral-domain-optical coherence tomography (SD-OCT, Heidelberg Engineering, Heidelberg, Germany) images pre-and postoperation and the presence of PIMH, as determined by SD-OCT.

The exclusion criteria were as follows: (1) presence of anamnestic data of previous eye trauma, (2) high myopia (axial length greater than 26.50 mm or refractive error more than 6.00 D), (3) rhegmatogenous retinal detachment with macular hole, (4) history of fundus disease, (5) low-quality SD-OCT image, which was defined as an image with no diameter measurement, (6) postoperative follow-up shorter than 4 months, (7) absence of comprehensive presurgery examination, and (8) glaucoma or other concurrent vision-limiting eye conditions were excluded.

### 2.3. Outcome measures

Baseline patient demographics include age, sex, ocular characteristics, mean preoperative minimum linear diameter (MLD) of the PIMH, and lens status (Table 1). All patients underwent ophthalmic examination, including Snellen best-corrected visual acuity (BCVA) test, SD-OCT scans, intraocular pressure measurement, slit-lamp biomicroscopy, fundus photography, and axial length measurement, pre-and postoperatively. All patients were followed up at outpatient clinics for at least 4 months postoperatively. Complications, such SiO emulsification and raised intraocular pressure (greater than 21 mmHg), were recorded.

**Table 1:** Baseline patient characteristics (76 patients, *n* = 76 eyes).

Factor	SiO (*n* = 21)	Air (*n* = 55)	*p value*
*Demographic characteristics*			
Age, years, mean ± SD (range)	60.8 ± 7.8 (43–76)	61 ± 7.0(45–80)	0.94
Sex, female, no.(%)	14 (66.7%)	40 (72.7%)	0.61
*Ocular characteristics*			
Mean preoperative BCVA, logMAR, mean ± SD	1.1 ± 0.4 (0.4–1.7)	1.2 ± 0.3 (0.5–2.0)	0.18
Mean preoperative MLD, μm, mean ± SD	605.6 ± 258.6 (177–1,040)	628.4 ± 247.1(256–1,363)	0.73
Duration between iMH and PIMH surgeries, months, mean ± SD	3.4 ± 3.2 (0.5–6.0)	5.4 ± 14.7(0.3–108)	0.53
Lens status, Phakic, no.(%)	19 (90.5%)	50 (90.9%)	0.91

MH, macular hole; SD, standard deviation; BCVA, best-corrected visual acuity; logMAR, logarithm of the minimum angle of resolution; MLD, minimum linear diameter. Mean ± SD is the mean diameter of the narrowest part of the hole ± standard deviation.

The primary outcome was anatomical success defined on OCT. We noted all available OCT examination data at the following time intervals: prior to revision surgery, post-revision surgery, and finally, at the most recent follow-up visit. According to the Manchester large macular hole study in which iMH size was the linear width across the narrowest point of the hole, we defined large PIMH as PIMH with a MLD of larger than 650 μm, and those in the 400–650 μm quartile were graded as medium PIMH (14). We measured PIMH using the available instruments (Spectralis [Heidelberg Engineering GmbH, Heidelberg, Germany]); diameters and widths were measured using the OCT caliper function (Cirrus HD software). OCT analysis was performed manually by one surveyor and the images were confirmed by one specialist, according to the benchmark mentioned above.

**Figure 1:**
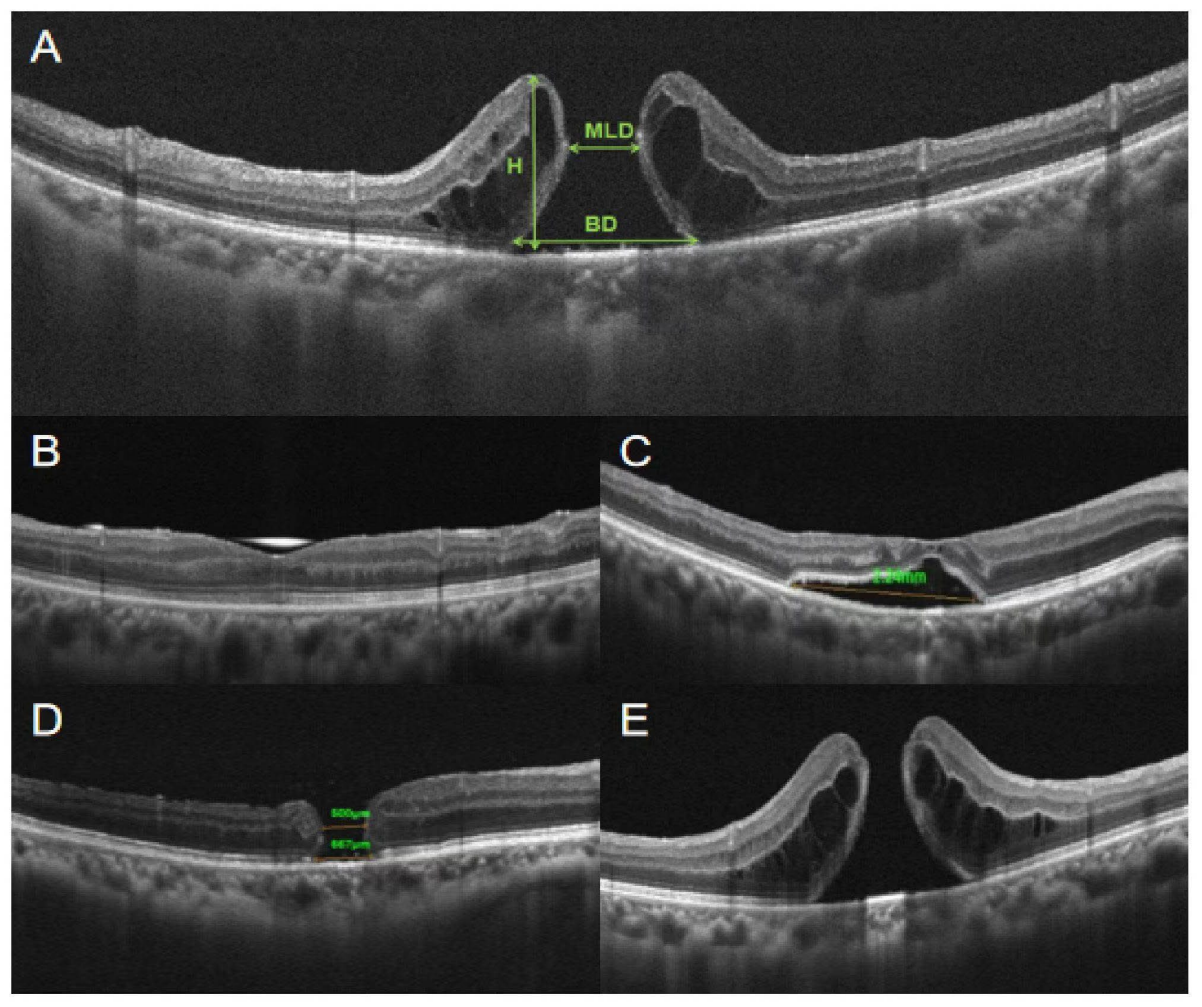
Diameters and features of PIMH. **(A)** Minimum linear diameter (MLD) and base diameter (BD) are parallel to the retinal pigment epithelium (RPE) at the nearest point of the retinal apposition, as described by Duker et al. (15). Both widths are measured as a line drawn roughly parallel to the RPE, Height (H) is defined as the distance between the highest edge of the hole and the RPE. **(B)** Flat-closed refers to complete contact between the edges of the hole, with complete coverage of the pigment epithelium layer and no subretinal fluid accumulation. **(C)** Elevated-closed refers to complete contact between the edges of the hole, with no exposure of the pigment epithelium but with a reservoir of subretinal fluid. **(D)** Flat-open refers to a defect of the retina, where PIMH edges attach to the RPE. **(E)** Elevated-open refers to a defect of the retina, where PIMH edges leave the RPE and are upturned.

Using OCT images, we measured MLD, base diameter (BD), and height (H) to describe the anatomical characters of PIMHs (Figure 1). Hole closure was defined as closure of an PIMH, without exposure of the retinal pigment epithelium (RPE), on OCT, in all radial scan meridians.

Secondary outcomes included functional BCVA and significant gain in BCVA. Snellen visual acuities were converted to the log of the minimum angle of resolution (logMAR) units for statistical analysis, and non-numeric values were changed as follows: count fingers = 1.7 Log MAR, hand movement = 2.0 LogMAR (20/2000 Snellen), light perception = 2.3 LogMAR, and no light perception = 3.0 LogMAR (20/20000 Snellen) (14). Significant gain in BCVA was defined as an increase (from baseline) in visual acuity of ≥2 lines on a Snellen chart, which is equivalent to an increase of ≥0.2 logMAR (16).

### 2.4. Surgical technique

Primary surgical techniques were performed by standard 25-or 27-gauge PPV, with or without the use of triamcinolone. Standard ILM peeling was performed in all cases after indocyanine green staining. Internal tamponade was given with a fluid-air exchange and an intraocular nonexpansile air tamponade. Reoperations were performed under local anesthesia, using either a 25-or 27-gauge system (Constel-lation^®^ Vision System; Alcon Laboratories, Fort Worth, TX, United States). In the SiO group, standard three-port PPV, followed by enlargement of the ILM peeling with adjunctive 0.125% indocyanine green staining, was performed by the same surgeon. Indocyanine green staining was performed to elucidate the extent of the original ILM peel. Care was taken as 25-gauge or 27-gauge forceps were used to ascertain the presence of residual perifoveal ILM or other additional membranes that required peeling. The fluid-air exchange was then repeated, with drainage of the preretinal fluid over the optic disc. Air was exchanged for SiO of 5,000 centistoke viscosity. Patients were instructed to maintain a face-down or prone position for 2 weeks postoperatively. Approximately 6 months after the surgery, the SiO was removed through a machine-independent method using a short infusion tube connected to a 10-mL syringe. Residual droplets of SiO were removed by using a flute needle to capture small oil bubbles in the vitreous cavity (17). All patients with phakic eyes underwent SiO removal combined with phacoemulsification and intraocular lens implantation simultaneously. In the air group, the three retina surgeons performed a similar procedure. Reoperation, performed with the standard three-port PPV, extended ILM peeling followed by fluid-air exchange and nonexpansile air tamponade. Patients were instructed to maintain a face-down or prone position for 3 days to 1 week postoperatively.

### 2.5. Statistical analysis

Statistical analyses were performed using SPSS 22.0 (SPSS for Windows, Chicago, IL). All continuous variables conformed to the normal distribution and were expressed as descriptive statistics, including total numbers, means, standard deviations, and percentages. Descriptive statistics were used to calculate demographic data. Means with standard deviation were presented where relevant. Paired *t*-test, Fisher’s exact test, and Bonferroni multiple comparison test were used as appropriate. Logistic regression was performed with the binary dependent variable of “open” or “closed” to assess the impact of the independent variables. A *p* value less than 0.05 was considered statistically significant. Confidence intervals were calculated to 95%.

## 3. Results

### 3.1. Characteristics of the patients

We enrolled 76 patients (21 patients [21 eyes] who underwent extended ILM peeling with SiO tamponade and 55 patients [55 eyes] who underwent extended ILM peeling and air tamponade) from January 2016 to June 2022 at Zhongshan Ophthalmic Center. Average age, sex ratios, ocular characteristics, duration between the iMH and PIMH surgeries, and base lens status were balanced between the two groups. The clinical characteristics of the patients at baseline are shown in Table 1.

### 3.2. Anatomic outcomes

In the SiO group, a total of 18 eyes of 21 patients (85.7%) achieved anatomical closure (Table 2), with 16 flat-closed and 2 elevated-closed types (Figure 1). All patients received their SD-OCT results on postoperative day one, and most PIMHs were closed. In the air group, 40 eyes (72.7%) achieved closure. There was no statistically significant difference between the two groups.

**Table 2:** Final anatomic outcome in the two groups.

Final anatomic outcome	Number (%)		*p* value
	SiO (*n* = 21)	Air (*n* = 55)	
Open	3 (14.3)	15 (27.3)	
Closed	18 (85.7)	40 (72.7)	0.37

*p* value is calculated using Fisher’s exact test.

**Figure 2:**
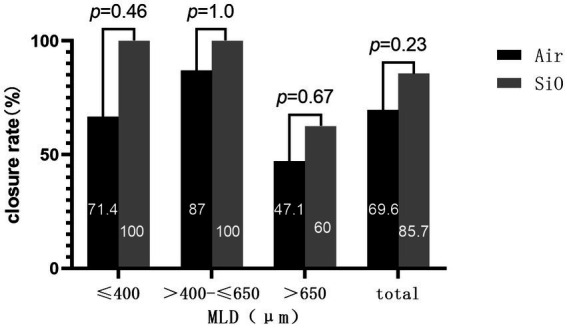
Anatomic outcomes of the two groups for different diameters.

We found that for all cases of MLD ≤650 μm, extended ILM peeling and SiO tamponade could produce stable anatomical closure success (100%). Further, when the MLD of PIMH was >650 μm, the success rate reduced notably (60.0%, Figure 2). For both groups, 650 μm (MLD) seemed like a cut-off point for closure rate (MLD ≤650 μm vs. MLD >650 μm: 88.4% vs. 52%, *p* = 0.002).

### 3.3. Effects on vision

In the SiO and air groups, vision significantly increased (*p* = 0.038 and *p* < 0.001), and the mean postoperative BCVA improved in the closed subgroup (*p* = 0.041 vs. *p* < 0.001; Table 3). The visual acuity of all patients in this series was poor before the intervention, but by the final follow-up examination, BCVA had improved in 13 eyes (61.9%), was stable in four eyes (19.1%), and had worsened in four eyes (19.1%) in the SiO group. In addition, in the SiO group, among the 18 patients who obtained anatomical closure success, a significant increase in visual acuity was observed in four patients.

**Table 3:** BCVA comparison based on anatomical outcomes in the SiO and air groups.

Macular status	SiO group			Air group		
	Mean BCVA at baseline, LogMAR	Mean BCVA at the final visit, logMAR	*p* value	Mean BCVA at baseline, logMAR	Mean BCVA at the final visit, logMAR	*p* value
Closed	1.11 ± 0.34 (0.6–1.7)	0.87 ± 0.40 (0.3–1.7)	0.041*	1.57 ± 0.09 (1.4–1.7)	1.05 ± 0.42 (0.5–1.6)	<0.001*
Open	1.17 ± 0.6 (0.5–1.7)	1.0 ± 0.30 (0.7–1.3)	0.7	1.57 ± 0.12 (1.5–1.7)	1.53 ± 0.06 (1.5–1.6)	0.68
All	1.12 ± 0.4 (0.52–1.70)	0.91 ± 0.40 (0.15–1.7)	0.038*	1.57 ± 0.10 (1.4–1.7)	1.14 ± 0.42 (0.5–1.6)	<0.001*

*Significant association (*p* value < 0.05).

### 3.4. Analysis of factors affecting PIMH closure

Previous studies have documented that the time between iMH surgery and PIMH surgery influences the anatomical closure rate (18). We also analyzed the duration between the two surgeries but did not get statistically significant results (OR, 1.03; 95% CI [0.94–1.13]; *p* = 0.54) (Table 4). Univariate regression analysis showed that MLD was the only parameter closely related to the closure rate (OR, 1.0; 95% CI (0.985–0.999); *p* = 0.03).

**Table 4:** Analysis of factors affecting PIMH fracture closure.

Factor (PIMH)	Odds Ratio (95% Confidence Interval)	*p* Value^†^
MLD	1.0 (0.985–0.999)	0.03 *
BD	1.0 (0.99–1.001)	0.15
H	1.0 (1.0–1.02)	0.805
MHI	0.0 (0.0–17.6)	0.12
THI	0.41 (0.02–8.82)	0.57
BCVA at baseline	0.71 (0.06–8.92)	0.79
Time duration between iMH and PIMH surgery	1.03 (0.94–1.13)	0.54
Age	0.96 (0.84–1.09)	0.515
Female gender	6.46 (0.81–51.34)	0.08
Tamponade	0.31 (0.01–8.75)	0.49
Binocular iMH	2.53 (0.26–25.03)	0.43

Macular hole index (MHI) is defined as the ratio of height to base diameter, that is MHI=H/BD. Tractional hole index (THI) is defined as the ratio of height to the MLD: THI=H/MLD.

^†^Univariate regression analysis, *n* = 75, *Significant association (*p* value < 0.05).

**Figure 3:**
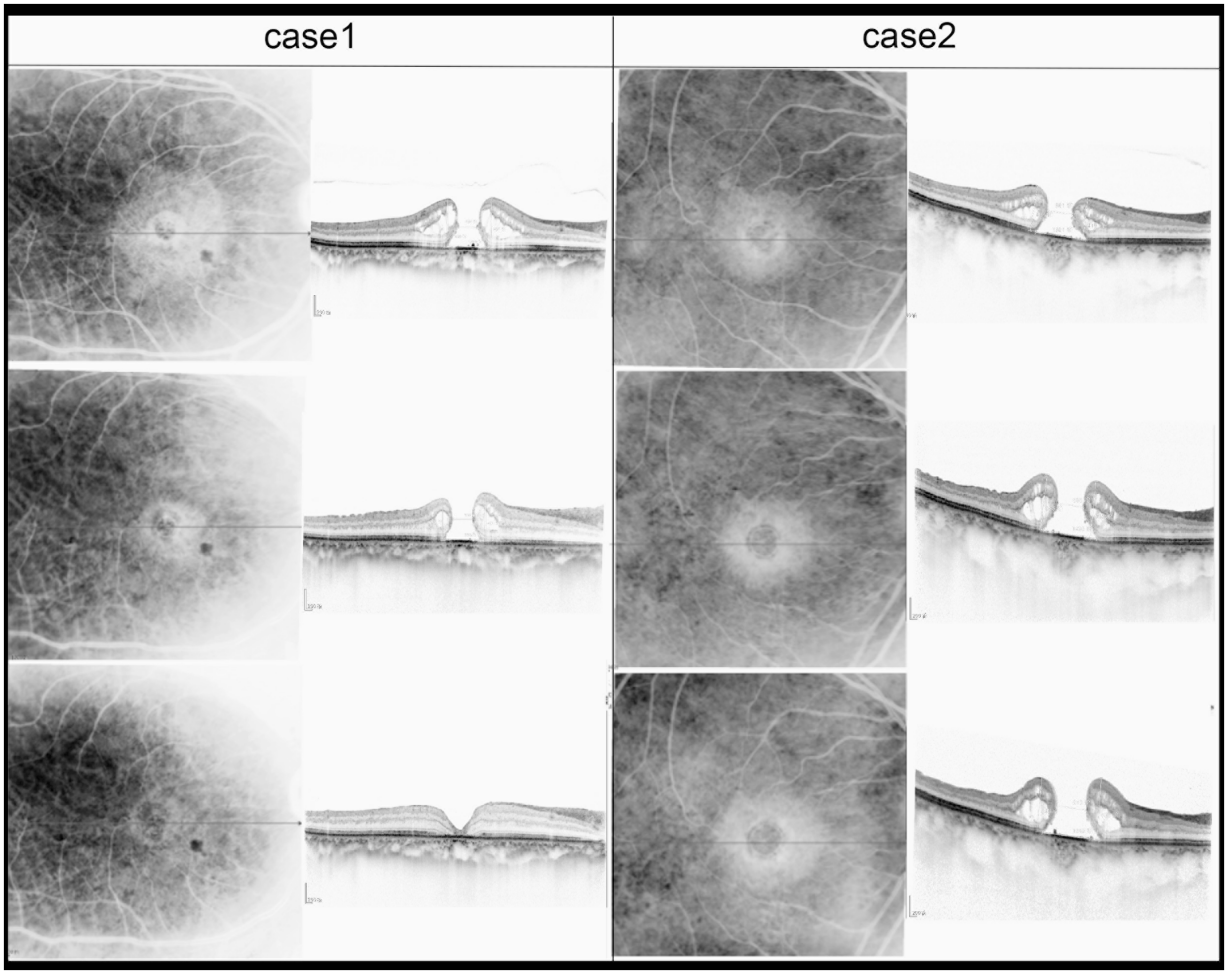
Sequential SD-OCT images of two patients were taken before iMH, 1 month after iMH surgery, and 1 month after PIMH surgery. MLD of PIMH of Case 2 is 686 μm. MLD of PIMH of Case 1 is 538 μm.

Another structural feature of PIMH we studied based on OCT findings was the elevated edge of the PIMH at baseline (Figure 3). We noted that 14 of 16 patients (87.5%) who had an PIMH with an elevated edge at baseline experienced PIMH closure after surgery, while four of five patients (80.0%) with a flattened PIMH edge experienced closure after the revision surgery. The difference between these groups of patients was non-statistically significant.

### 3.5. Follow-up and postoperative complications in the SiO tamponade series

Overall, 19 eyes (90.5%) were phakic at baseline, with the remaining eyes having a history of cataract extraction, including intraocular lens implantation. Of the patients with phakic eyes, 16 patients (88.9%) underwent advanced cataract extraction, and a majority (93.8%) underwent SiO removal combined with cataract extraction within 1 year of the repeat surgery (mean 6.2 ± 1.3 months). One patient developed high intraocular pressure after SiO tamponade, which resolved after pharmacotherapy. No other adverse events, such as SiO emulsification or cystoid macular edema, were observed during the follow-up. Two patients in the SiO group experienced subretinal fluid accumulation during the follow-up period, but at the end of the follow-up period, the fluid level had decreased and visual acuity had improved in both patients. In the air group, one patient experienced retinal detachment 9 months after PIMH surgery. The details of three patients that did not achieve anatomical closure success are listed in Table 5.

**Table 5:** Details of three unclosed cases after SiO tamponade (*n* = 3).

MHs that opened in the final examination	Sex	Age	Time interval between MH surgeries (months)	Follow-up Duration (months)	Preoperative BCVA, logMAR	Cataract Treatment	Preoperative MLD, μm	Postoperative MLD, μm	Preoperative MLD, μm (mean ± SD^*^)	Postoperative MLD, μm (mean ± SD^*^)	*p* value^†^
Case 1	Female	66	2	10	1.7	IOL	970	692	903.67 ± 90.941	776.33 ± 142.620	0.444
Case 2	Female	66	6	11	1.3	NO	941	696			
Case 3	Female	55	1	7.5	0.5	YES	800	941			

MH, macular hole, SD, standard deviation. *mean ± SD is the mean diameter of the narrowest inner ± standard deviation. **All MHs obtained flat-open closure at the final examination after SiO removal, thus, postoperative narrowest inner diameter = postoperative basal diameter †By paired *t*-test. Significant association (*p* value < 0.05).

## 4. Discussion

Failure of the primary surgery and persistent iMH is the most common complication of iMH surgery. Successful secondary closure of iMH is probably influenced by multiple factors. Several factors have been linked to the failure of primary iMH surgery, including residual traction from ILM, poor patient compliance with proper positioning, and size of iMH > 400 μm. Whether the choice of intraocular gas or SiO as a surgical tamponade in the second iMH surgery affects surgical success is unclear.

We found notable facts: live retinal tissue studies have suggested that the retina is soft and actively mechanoresponsive, approximately 100 times more compliant than soft silicone rubber (19, 20). Broad peeling of a taut ILM may enhance this compliance by relaxing the intrinsically elastic retinal tissue (21). Further, past clinical experience has indicated that prolonged tamponade would lead to a higher rate of effective PIMH closure (18). Thus, we hypothesized that broad peeling of a taut ILM or long-term tamponade, such as using SiO, may be effective. In this study, the surgery of choice for PIMHs was extended ILM peeling and SiO tamponade. We added a matched cohort of patients who underwent air tamponade for its involvement in short-term tamponade and extended ILM peeling to get more information. To the best of our knowledge, this is the first study to evaluate the benefit of these two procedures for PIMH.

The anatomic closure rate in our SiO group was 85.7%, similar to recently reported results in the literature. Conversely, the anatomic closure rate in our air group was 72.7%, which was worse than in a previous study (22, 23). We also noticed a trend toward a higher anatomical closure rate in the SiO group, although this difference was not statistically significant. Conclusively, using SiO and extended ILM peeling does not seem to improve the anatomical and functional outcome of surgery. However, it is difficult to compare the results of our group with those of previously published reports that used SiO because of differences in the MLD basement or in the definition of anatomic closure among the studies. This research employed the evaluation method posited by Kang and colleagues (24); type 1 closure corresponds to flat-closed and elevated-closed, and type 2 closure corresponds to flat-open and elevated-open. Moreover, flat-closed and elevated-closed were defined as forms of an-atomical closure. MLD ≤400 μm has been considered the safest range for anatomical closure. We found that for all cases of MLD ≤650 μm, extended ILM peeling and SiO tamponade could produce stable anatomical success.

In our series, among all cases of anatomical closure, most cases of PIMH were associated with closure on the postoperation first day (16/18, 88.9%). Thus, we can hypothesize that SiO supplies strong tension for aggregating hole margins, and using SiO of 5.000 centistokes of viscosity may elucidate this phenomenon. Because closure detection is via OCT, we could not verify the closure rate in the air tamponade group on the postoperation first day. Therefore, closure speed could not be compared between the two groups.

Functional outcome is typically more important for patients than anatomical closure, although it could be extremely difficult for PIMH. SiO tamponade has been considered less effective in improving vision than gas tamponade. A 2021 study conducted by Li et al. used SiO tamponade in 33 patients with PIMH and reported that 1.00 logMAR (0.60–1.00) at baseline was significantly improved to 0.65 logMAR (0.49–1.00; *p* = 0.010) at the final examination (25). In our study, more than half of the patients in the SiO group experienced vision improvement, and among the 18 patients who achieved anatomic success, four patients obtained a significant increase in visual acuity. Notably, cataract surgery at the time of SiO removal positively influences visual outcomes. Besides, we noted non-improvement in vision in the group that did not achieve anatomical closure. We extensively analyzed the three cases of failed closure and non-improvement in vision after SiO tamponade (Table 5) and only noticed a larger preoperative MLD.

It was important for us to determine the factors that influence operative outcomes. We analyzed factors affecting PIMH fracture closure (Table 4). Although the number of cases in our study may be a limitation, the consistent use of the two different types of surgery for PIMH treatment showed that the final closure rate depends on the MLD of PIMH. We could not find similar studies on PIMH, which may be attributable to the low morbidity associated with PIMH. However, in the iMH field, Chhablani et al. (26) performed a retrospective study of 137 eyes of 137 patients who underwent iMH repair and reached the same conclusion—the minimum diameter between the hole edges and the longest diameter of the hole is the best predictors of hole closure.

There are some disadvantages of SiO tamponade, the most important one being the need for additional surgery. However, cataract formation after PPV, whatever the form of tamponade used, is a well-known and inevitable complication of the surgery. A study reported up to an 81% cataract risk after 6 months and 98 and 100% risk at one and 2 years, respectively, after vitrectomy (27). The incidence of cataract surgery in the present study is comparable to those of other studies investigating cataract occurrence after macular hole surgery (21, 28). Therefore, all our phakic patients in the SiO group proposed to undergo cataract surgery combined with SiO removal. One previous study employed a surgical approach—cataract and SiO removal—and compared them in combination with the two steps, respectively (29). Their results suggest similar visual outcomes and complication rates in both groups. In our cohort, we combined cataract extraction and SiO performed at 6.2 ± 1.3 months after the PIMH operation and observed faster visual rehabilitation with no complication at the final follow-up.

The limitations of this study include the small size and the relatively short follow-up period. For some special reasons in China, we did not get a gas tamponade group for comparison during our research. Table 3 only shows 16 follow-up records in the air group because of the impact of the COVID-19 pandemic. An unforeseen selection bias may have existed for participants who accepted silicone filling because of the surgeon’s experience. Generally, PIMHs are more complex, difficult to treat, and filled with SiO. Furthermore, previous studies compared SiO-filled eyes with gas-filled eyes, and variable postoperation BCVA was noted, suggesting that direct macular toxicity related to SiO may adversely affect visual acuity, although there is little supportive evidence. Further studies involving larger patient numbers and longer follow-ups are warranted.

## 5. Conclusion

In summary, in terms of anatomical and functional outcomes, extended ILM peeling combined with SiO or air tamponade is effective in treating PIMH. Moreover, though not statistically significant in the present study, the anatomic closure rate seems better for silicone-operated eyes than for air-operated eyes in the real world. MLD is the best predictor of PIMH closure, with MLD ≤ 650 μm associated with a significantly higher closure rate. The information in this article can help surgeons and PIMH patients decide whether and how to proceed with a second surgery.

## Data availability statement

The original contributions presented in the study are included in the article/supplementary material, further inquiries can be directed to the corresponding authors.

## Ethics statement

Written informed consent was obtained from the individual(s) for the publication of any potentially identifiable images or data included in this article.

## Author contributions

YS: conceptualization. YS and LW: data curation. LF: formal analysis. LF and XH: methodology. YL: project administration. YS, YL, and DF: resources. DF: visualisation. YS: writing – original draft. YW, TZ, and SZ: writing – review & editing. All authors contributed to the article and approved the submitted version.

## Funding

This study was supported by grants Shenzhen Science and Technology Program (KCXFZ20211020163813019) and the Sanming Project of Medicine in Shenzhen (SZSM202011015).

## Conflict of interest

The authors declare that the research was conducted in the absence of any commercial or financial relationships that could be construed as a potential conflict of interest.

## Publisher’s note

All claims expressed in this article are solely those of the authors and do not necessarily represent those of their affiliated organizations, or those of the publisher, the editors and the reviewers. Any product that may be evaluated in this article, or claim that may be made by its manufacturer, is not guaranteed or endorsed by the publisher.
